# Solid-Phase Spectrophotometric Analysis of 1-Naphthol Using Silica Functionalized with *m*-Diazophenylarsonic Acid

**DOI:** 10.1186/s11671-016-1356-2

**Published:** 2016-03-15

**Authors:** Nataliya Zaitseva, Sergei Alekseev, Vladimir Zaitsev, Viktoria Raks

**Affiliations:** Department of Analytical Chemistry, Faculty of Chemistry, Taras Shevchenko National University of Kyiv, Volodymyrska Street, 64/13, Kyiv, 01601 Ukraine

**Keywords:** 1-Naphtol, Solid-phase spectrophotometry, Solid-phase extraction, Azo coupling reaction, Functionalized silica

## Abstract

The *m*-aminophenylarsonic acid (*m*-APAA) was immobilized onto the silica gel surface with covalently grafted quaternary ammonium groups via ion exchange. The diazotization of ion-bonded *m*-APAA resulted in a new solid-phase spectrophotometric reagent for detection of 1-naphtol in environmental water samples. The procedure of solid-phase spectrophotometric analysis is characterized by 20 μg L^−1^ limit of detection (LOD) of 1-naphtol, up to 2000 concentration factor, and insensitivity to the presence of natural water components as well as to 30-fold excess of phenol, resorcinol, and catechol.

## Background

Highly toxic and mutagenic derivatives of naphthols and aminonaphthols constitute an important class of environmental pollutants [1, 2]. Most of the naphthols appear in the environmental water due to the biodegradation of pesticides and azo dyes [3, 4]. For example, environmental decomposition of carbaryl (1-naphthyl-*n*-methylcarbamate), which is widely used as an insecticide, gives 1-naphthol [3], whereas common azo dyes give harmful pollutant amino-2-naphthol [4].

Usually, the naphthols are analyzed by high-performance liquid chromatography [5], flow-injection analysis with further photometric detection [6], fluorimetry [7–9], phosphorimetry [10], and by immunosensors [11]. Fluorimetric, phosphorimetric, and immunosensor methods require complicated sample pre-treatments. Photometric method is much more reliable for infield analysis; however, its sensitivity is insufficient for most of the environmental cases [9]. Therefore, the pre-concentration of naphthol derivates by means of liquid-liquid or solid-phase extraction (SPE) is commonly used in sample preparation prior to photometric determination [9, 10, 12–14]. Several approaches for selective SPE were proposed in literature: (1) pre-concentration of 1-naphthol derivate on silica with immobilized Co^3+^ [12] or polyurethane foam [13]; (2) formation of inclusion complexes of naphthols and 3-Br-1-propanol in β-cyclodextrine [10]; (3) microextraction of 1-naphthol on a glass capillary modified with polydimethylsiloxane-divinylbenzene [14].

The selectivity of pre-concentration procedure appeared the major concern for environmental samples analysis. Developments in this area are still needed due to only few reports on selective adsorbents preparation [15–17]. It is even more challenging to develop selective solid-phase reagent allowing the naked-eye monitoring of naphthol contaminations in environmental water.

The reaction of azo coupling, taking place between diazonium salts (DS) and aromatic amines, phenols, or naphthols, results in deep-colored products (azo dyes). Therefore, it is widely used for photometric analysis of organic pollutants [18–20]. However, due to modest selectivity and insufficient sensitivity, the azo coupling is not fully applicable on environmental samples. This limitation can be overcome if the procedures of pre-concentration and analysis are combined in one solid-phase analytical reagent (SPAR). To develop such a SPAR, we propose immobilization of azo coupling reagent on the silica gel surface.

The present work aimed to develop a new SPAR for selective pre-concentration of 1-naphthol traces from water samples. SPAR changes its color, with no additional reagent added, as the pollutant’s concentration increases up to its maximum allowed concentration (MAC). To evaluate the level of the pollutant in a water sample, a UV–vis diffuse reflection spectroscopy can be used. The SPAR, which is introduced in this work, is based on *m*-aminophenylarsonic acid (*m*-APAA), immobilized on silica with grafted quaternary ammonium anion exchanging groups. The azo coupling between 1-naphthol and the DS, formed by diazotation of immobilized *m*-APAA, ensures high efficiency of the pollutant pre-concentration and selectivity of SPE; intense color of azo product means low detection limit.

## Methods

### Apparatus

Measurements of pH and electrode potentials were performed by a laboratory ion-meter I-160M (Antech, Belarus). A peristaltic pump 2132 LKB Bromma was used to set liquid flow rates in dynamic adsorption experiments. The UV–vis absorbance spectra of solutions were measured by an UV-2401 PC (Shimadzu) spectrophotometer in 220–900-nm range; the diffuse reflection UV–vis (DR-UV) spectra of solids were recorded by a CS-9301 PC densitometer (Shimadzu).

### Reagents and Materials

Reagent grade chemicals (Merck) were used without further purification. *m*-Aminophenylarsonic acid was obtained from *m*-nitrophenylarsonic acid by its reduction with ferrous sulfate in water solution according to the literature method [21]. The рН values of solutions were maintained by buffer prepared from 0.05 mol∙L^−1^ sodium tetraborate and hydrochloric acid. The concentration of *m*-APAA and its diazotized product was measured by spectrophotometry according to [22]. Concentrations of 1-naphthol, resorcinol, and catechol were measured by spectrophotometry after the reaction with 4-aminoantipyrin according to [23].

### Synthesis of Trimethyl(3-Trimethoxysilylpropyl)Ammonium Iodide (TMPA)

**Scheme 1:**
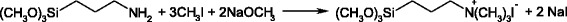
The reaction scheme for the synthesis of trimethyl(3-trimethoxysilylpropyl)ammonium iodide

Syntheses of TMPA and silica with covalently immobilized TMPA (SiO_2_–TMPA) were performed in anhydrous solvents under Ar. The TMPA was obtained according to Hoffman reaction (Scheme 1).

For this, 0.01 mol of 3-aminopropyltrimethoxysilane was mixed with 0.04 mol of methyl iodide in 50 mL of methanol at room temperature. After during 1 h time 0.02 mol of CH_3_ONa in 10 mL of CH_3_OH was added dropwise to the reaction mixture followed by stirring for 20 h. After the solvent was evaporated in vacuum (0.1 mmHg), white residual, giving 0.4 t(2H), 1.65–1.8 m(2H), 3.1–3.3 m(11H), 3.51 s(9H) ppm signals in 1H NMR spectrum in CDCl_3_ solution, was used in SiO_2_–TMPA with no further purification.

### Synthesis of Silica with Grafted Anion Exchange Groups (SiO_2_–TMPA)

Prior functionalisation, silica gel carrier was annealed for 8 h in air at 500 °C. Activated on such way, silica gel (8 g) was immersed to 50 mL of CH_3_CN and TMPA was added. The suspension was stirred during 15 h at 80 °C and then the silica was filtered, washed with CH_3_CN in the Soxhlet extractor for 5 h, and dried in vacuum. Obtained organo-silica was immersed to saturated KBr aqueous solution for 5 min, washed with this water by decantation, and finally dried on air at 105 °С. The concentration of alkylammonium groups immobilized on SiO_2_–TMPA was determined from argentometric titration as 552 μmol g^−1^.

### Synthesis of Silica with Immobilized *m*-АPAA (SiO_2_–*m*-АPAA)

Aqueous solution of *m*-АPAA (25 mL, 1.2 × 10^−2^ mol L^−1^) was passed through the column (*d* = 5 mm, *h* = 100 mm) filled with 0.5 g of SiO_2_–TMABr with 0.5 mL min^−1^ flow rate. Obtained SiO_2_–*m*-АPAA was washed with distilled water and dried at room temperature for 24 h. The concentration of immobilized *m*-АPAA groups was calculated as a difference between the concentration of *m*-АPAA in solution before and after passing through the column and the concentration found in the distilled water.

### Synthesis of Silica Modified with *m*-Diazophenylarsonic Acid (SiO_2_–DS) and its Hydrolytic Stability

Diazotization of immobilized *m*-APAA was performed according to reported procedure [22]. For this, 50 mg of SiO_2_–*m*-АPAA was mixed with 5 mL of 5 × 10^−2^ mol L^−1^ NaNO_2_ and 5 mL of 0.5 mol L^−1^ HCl. The mixture was shook for a while. The adsorbent was washed quickly with distilled water followed by buffer solution (pH = 8). Since the diazonium salts are thermally unstable, all procedures were carried out below 5 °C.

### Adsorption Studies of SiO_2_–DS

The adsorption capacity of SiO_2_–DS to phenolic compounds was determined in dynamic conditions. Aqueous solutions of phenols (5 × 10^−4^ mol L^−1^) having pH 8 were passed (0.2 mL min^−1^) through a column packed with 300 mg of SiO_2_–DS. The concentration of phenols was determined in each 2 mL portion of the effluent. The adsorption capacity of SiO_2_–DS was calculated by Eq. (1):

1 $$ \alpha =\frac{V}{g}{\displaystyle \sum_{i=1}^k\left({C}_0-{C}_i\right)}, $$

where *k* is the number of solution portions passed through the column, *g* is the mass of adsorbent in the column, *C*_0_ is the initial concentration of phenolic compound in the solution, *C*_*i*_ is its concentration in each portion of the effluent, and *V* is the volume of effluent portion.

The adsorption kinetics was studied in static conditions. Fifty milligrams of SiO_2_–DS was mixed with 25 mL of 2 × 10^−5^ mol L^−1^ solution of the phenolic compound at pH 8 and shaken during fixed time interval (1–10 min). The solid phase was filtered, washed with pH 8 buffer, and dried at room temperature. The solution was analyzed for phenol contents while the solid phase was used to record the UV–vis spectrum.

### Solid-Phase Spectrophotometric Detection of 1-Naphthol

To make a calibration curve, portions of aqueous 1-naphthol solutions (95 mL) with a concentration in a range 0–2 mg L^−1^ were mixed with 5 mL portions of buffer solution (pH = 8). After cooling down into the ice bath, these solutions were added to as-prepared 50 mg portions of SiO_2_–DS and vigorously stirred for 5 min. The solid phase was filtered, washed with water, and dried at room temperature. The DR-UV spectra were recorded and presented as Kubelka-Munk function (*F*(*R*) = (1 − *R*)^2^/2*R*) giving the intensity of spectrum signals which is directly proportional to concentration of absorbing species in the solid phase.

To analyze natural water samples, 95 mL samples (as received or spiked with 100 μg L^−1^ of 1-naphthol) without any pretreatment were mixed with 5 mL portions of buffer (pH = 8) and treated as described above.

## Results and Discussion

Several strategies of the DS immobilization on the silica gel surface are possible: covalent grafting [24, 25], physical adsorption, and ion exchange binding. Due to low stability of diazonium salts in solution and even in immobilized state [26], it is desirable to have fast and simple procedure for the SiO_2_–DS preparation, which is looking problematically for covalent grafting. The physisorption of organic reagents also seems doubtful, particularly due to desorption of DS at the stage of adsorbent application. To avoid the above drawbacks, the ion exchange approach is likely to be helpful; furthermore, silica-based anion and cation exchangers are thoroughly studied [27] and some of them are commercially available.

**Scheme 2:**
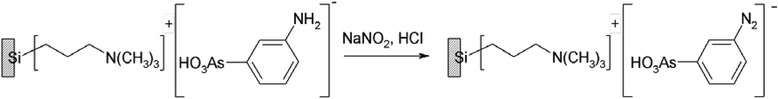
The reaction scheme for the diazonium salt immobilization on the silica gel surface

Due to the instability of diazonium salts in solution as well as in the immobilized state, a two-stage immobilization approach was used for preparation of SiO_2_–DS. At the first stage, a stable precursor of diazonium salt (*m*-APAA) was immobilized onto the surface of silica with covalently grafted quaternary ammonium groups (Scheme 1). To obtain the SiO_2_–DS, amino groups of immobilized *m*-APAA were diazotized by treatment with a standard diazotizing mixture (HCl + NaNO_2_, see Scheme 2).

### Synthesis of SiO_2_–APAA

**Fig. 1:**
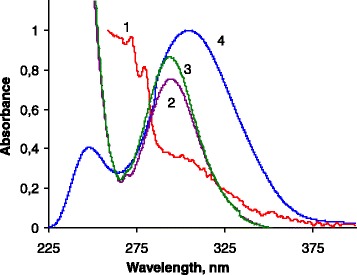
UV–vis absorption spectra of 5 × 10^−4^ M solution of *m*-APAA at different рН: (*1*) 2.0, (*2*) 4.0, (*3*) 9.0, and (*4*) DR-UV spectrum of SiO_2_ *–*APAA

The *m*-АPAA molecule contains acidic groups of intermediate strength (−AsO_3_H_2_) and weakly basic groups (−NH_2_). That is why it can exist in aqueous solutions as cationic (H_3_A^+^), neutral (H_2_A), and two anionic (HA^−^ and A^2−^) forms depending on the pH. Unfortunately, no exact values of the acidity constants were found in the literature for *m*-APAA. However, the isoionic pH values (i.e., pH_*i*_ = (pK_1_ + pK_2_)/2 which corresponds to maximum concentration of H_2_A and equal concentrations of H_3_A^+^ and HA^−^) are equal to 3.00 for *ortho-* and to 3.15 for *para*-APAA isomers, respectively [28]. These values allow us to assume that in aqueous solutions *m*-APAA can be either in its cationic H_3_A^+^ (pH ≤ 2) or anionic form (pH ≥ 4). Difference in the UV–vis absorption spectra of the *m*-APAA solutions at different pH values (Fig. 1) is probably caused by deprotonation of the amino group (formation of the auxochrome results in a spectral maximum redshift), which confirms ionization of the *m*-APAA at pH ≥ 4.

As the silica carrier is not stable at the pH >9 [29] and the *m*-АPAA exists in a protonated form at pH < 2, the ion exchange immobilization of *m*-АPAA on the SiO_2_–TMABr was studied in the pH range of 2–9. Maximum adsorption capacity to *m*-APAA (*С*_L_) is equal to 520 ± 10 μmol/g at pH = 4.0. This value corresponds to more than 94 % conversion of surface bromide salt (*С*_L_ = 550 ± 10 μmol/g according to argentometric titration data) to *m*-АPAA salt. Lower adsorption capacities found at other studied pH values may be explained by incomplete deprotonation of *m*-APAA at lower pH and the presence of doubly charged *m*-APAA anions at higher pH. That is why the SiO_2_-APAA for all further experiments was prepared at pH = 4. The position of absorption maximum in the DR-UV spectrum of SiO_2_–APAA (300 nm, see Fig. 1) coincides with the position of maximum in the solution UV–vis spectra of *m*-APAA anionic forms. This fact additionally confirms that the ion exchange immobilization of *m*-APAA on SiO_2_–TMABr occurs according to Scheme 1.

### Synthesis and Properties of SiO_2_–DS

The SiO_2_*–*DS was obtained by a reaction of SiO_2_–APAA with NaNO_2_ in acidic media (Scheme 2). However, the diazonium salt tends to desorb in these conditions. As it can be seen from Table 1, the degree of DS desorption (ratio of diazonium salt quantity, found in the effluents after addition of extra NaNO_2_ and HCl and 15 min equilibration according to [22] and quantity of *m*-APAA in initial modified silica) increased significantly with the diazotation time and especially with HCl concentration. That is why in all further experiments, the diazotation was performed in optimized conditions, during 1 min, by 0.25 mol L^−1^ HCl, which resulted in only 5 % of reactant desorption. Importantly, no diazonium salt was detected in a rinsing buffer solution with pH 8; hence, the weakly alkaline media inhibits the DS desorption. Value of the concentration of active diazonium groups in SiO_2_–DS can be estimated from the value of its adsorption capacity to phenolic compounds, as it is discussed below.

**Table 1:** Conditions of SiO_2_ *–*APAA diazotization and degrees of the DS desorption: *С*_L_(SiO_2_ *–*APAA) = 520 μmol g^−1^, *m* (SiO_2_ *–*APAA) = 0.05 g, *V*(HCl) = *V*(NaNO_2_) = 5 mL, *t* = 0–4 °C

*С*_HCl_ (mol L^−1^)	Time of treatment (min)	Desorption degree (%)
0.25	1	5
0.25	2	31
0.5	1	99

### Interaction of SiO_2_–DS with Phenolic Compounds

**Fig. 2:**
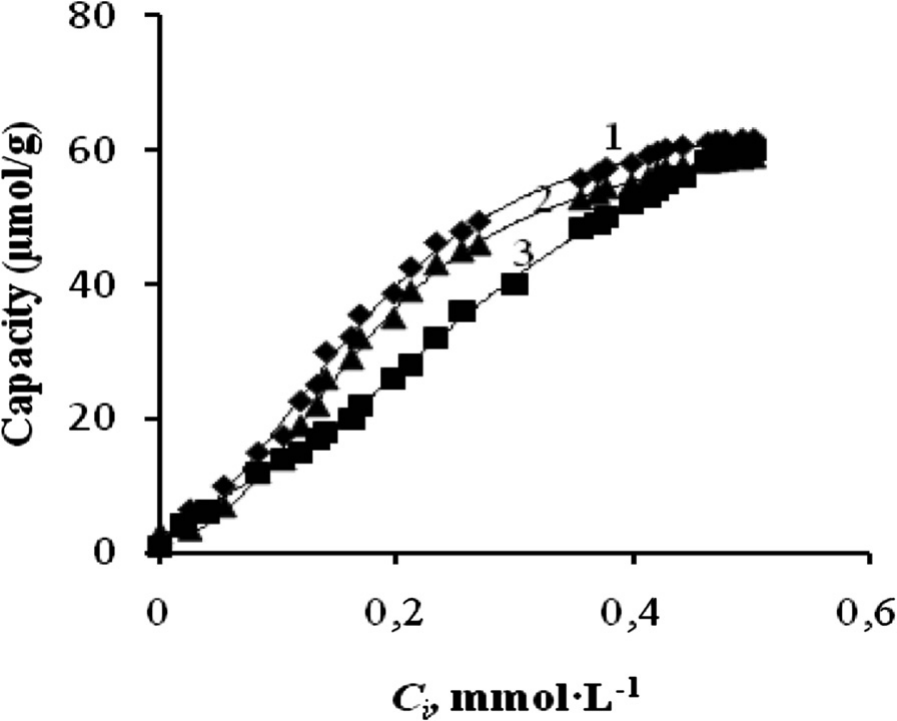
Dynamic adsorption isotherms (adsorption vs. concentration of phenolic compound (*C*_*i*_) in the effluent) of (*1*) 1-naphthol, (*2*) 2-naphthol, and (*3*) resorcinol on SiO_2_ *–*DS. (SiO_2_ *–*DS) = 300 mg, *C*_0_ = 0.5 mmol L^−1^, pH = 8, *υ* = 0.2 mL min^−1^

Common phenolic compounds are active in azo coupling reaction in the pH range 5–10. As the silica gel matrix becomes unstable at рН > 9, and the DS desorption takes place in the acidic media, the adsorption of phenolic compounds on SiO_2_*–*DS was studied at pH 8.0. Dynamic adsorption isotherms (Fig. 2) were obtained in the conditions, which are typical for the SPE applications.

**Scheme 3:**
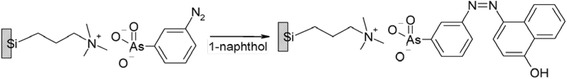
The reaction scheme for the formation of immobilized azo dye

Initial light-yellow color of the SiO_2_*–*DS changes to purple after interaction with phenolic compounds, confirming the formation of immobilized azo dye according to Scheme 3. At low loadings of phenolic compounds, nearly linear increase of adsorption with their concentration in effluent is observed, whereas at higher loadings, the isotherms come to saturation, corresponding to 60 μmol g^−1^ (Table 2). Probably, this value corresponds to the concentration of active diazonium groups on SiO_2_*–*DS. At the same time, no coloration of the effluent was observed, so no desorption of the DS as well as the azo dyes takes place under dynamic adsorption experiments.

**Table 2:** Adsorption capacities and dynamic distribution coefficients of SiO_2_ *–*DS toward phenolic compounds: *m* (SiO_2_–DS) = 0.05 g, *C*_0_ = 5 × 10^−4^ mol L^−1^, pH = 8, *υ* = 0.2 mL min^−1^

Compound	Adsorption capacity, μmol g^−1^	Distribution coefficient (*k*), g L^−1^
1-Naphthol	62	200
2-Naphthol	60	170
Resorcinol	59	190
Phenol	0 ± 2	–

Surprisingly, the phenol did not adsorb on SiO_2_*–*DS in noticeable amount under the selected conditions (pH 8); total adsorption capacity did not exceed 2 μmol g^−1^ and no significant coloration of SiO_2_*–*DS was observed. Such a specificity of SiO_2_*–*DS to polyhydroxy (resorcinol) and polyaromatic (naphthols) phenols can be explained by their higher activity in the azo coupling reaction comparing to the phenol. This last one can be adsorbed by SiO_2_*–*DS but in different conditions: involving higher pH and wider contact time span.

High values of adsorption capacities and dynamic distribution coefficients (in a range of 10^2^ g L^−1^, see Table 2) of SiO_2_*–*DS to 1-naphtol, 2-naphtol, and resorcinol enable its application for pre-concentration of those pollutants from environmental water samples. For example, MAC of 1-naphthol in drinking water was reported as 100 μg L^−1^ [30]. SiO_2_*–*DS packed (0.1 g) in SPE cartridge can be used for pre-concentration of 1-naphthol from approximately 250 mL of water solution having <5 MAC of 1-naphnol. The value of MAC for 1-naphthol in drinking water is 100 μg L^−1^. It means that 5 MAC values are equal to 500 μg L^−1^. Therefore, 250 mL of such a solution contains 125 μg of 1-naphthol. Adsorption capacity of SiO_2_*–*DS is 6700 μg g^−1^, so in case of SPE cartridge packed with 0.1 g of the SiO_2_*–*DS, the capacity is 670 μg. The ratio of the SPE capacity to the quantity of 1-naphtol in 250 mL is 670 μg:125 μg = 5,36. Therefore, under these conditions, the actual amount of the pollutant in a solution will be five times less than the adsorption capacity (6700 μg g^−1^).

### Diffuse Reflectance UV Spectra of Immobilized Azo Compounds and 1-Naphthol Detection

**Fig. 3:**
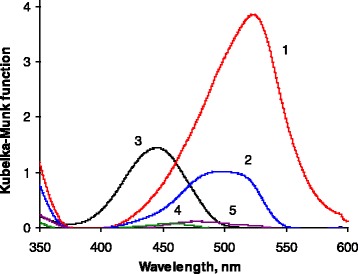
Diffuse reflectance UV–vis spectra of SiO_2_ *–*DS after treatment with: (*1*) 1-naphthol, (*2*) 2-naphthol, (*3*) resorcinol, (*4*) catechol, and (*5*) phenol. The experimental conditions were the same for all phenols: *m* (SiO_2_ *–*DS) = 50 mg, *C*_0_ = 2 × 10^−5^ mol L^−1^, *V* = 25 mL, pH = 8, *τ* = 10 min

High affinity of the SiO_2_*–*DS to active phenols and bright color of the reaction products make this adsorbent prospective to develop solid-phase analytical reagent (SPAR) for DR-UV spectrophotometric determination of these substances. Figure 3 demonstrates the diffuse reflectance UV–vis spectra of SiO_2_*–*DS after treatment with different phenolic compounds. As it was expected from the dynamic adsorption data, bright coloration of the adsorbent and corresponding intense bands in the visible spectrum range were observed only for active phenolic compounds: 1-naphthol (*λ*_max_ = 530 nm), 2-naphthol (*λ*_max_ = 490 nm), and resorcinol (*λ*_max_ = 440 nm), whereas for less active phenol and catechol, the adsorbent coloration was moderate. Partial overlapping of the spectral bands of different azo dyes should result in significant overestimation of 1-naphtol concentration, determined by developed SPAR in the presence of 2-naphtol, resorcinol, or other azo coupling active compounds, whereas the influence of less active phenol and catechol should be much lower.

**Fig. 4:**
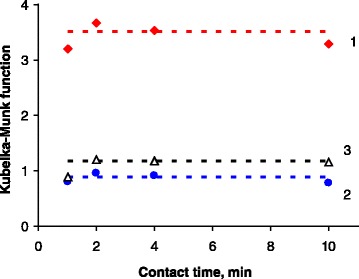
Dependencies of Kubelka-Munk function (at *λ*_max_) from the contact time of SiO_2_ *–*DS with: (*1*) 1-naphthol, (*2*) 2-naphthol, and (*3*) resorcinol. The intermediate values in 2–10 min range are indicated by *dotted lines. m* (SiO_2_ *–*DS) = 50 mg, *C*_0_ = 2 × 10^−5^ mol L^−1^, *V* = 25 mL, pH = 8

To find the optimal time of interaction between SiO_2_–DS and phenolic compounds, time dependencies of the analytical signal (i.e., the value of Kubelka-Munk function at *λ*_max_) were studied (Fig. 4). For the reaction times ≥2, the intensity of signal appeared nearly constant for all studied phenols, indicating high rates of their azo coupling according to Scheme 3. That is why 5 min equilibration time is sufficient for the formation of azo dye on the SiO_2_ surface, and this time interval was used in all further experiments.

As it can be seen from Figs. 3 and 4, the product of SiO_2_–DS interaction with 1-naphtol demonstrates the most intense color among all studied phenolic compounds, probably due to the highest extinction coefficient of the corresponding azo dye. Therefore, the highest sensitivity of 1-naphtol analysis procedure should be achieved. This factor together with the importance of 1-naphtol as an environmental pollutant allowed us to choose this compound as an analyte for further studies.

To study the effect of analyte dilution, samples of different volumes (25 mL–1 L) containing the same amount of 1-naphtol (0.5 μmol) were interacted with SiO_2_–DS (50 mg) in the conditions described above. Values of the analytical signal (*F*(*R*) at 530 nm) were compared. Variation of the solution volume in the 25–200-mL interval (500–4000 mL g^−1^ ratio of the solution volume to adsorbent mass, i.e., concentration factor) does not influence significantly on the signal; however, higher volumes result in its significant decrease (62 % from the initial value for 400 mL and 29 % for 1 L of solution).

**Fig. 5:**
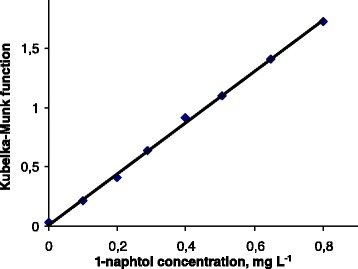
Calibration plot for 1-naphthol detection. *m* (SiO_2_ *–*DS) = 50 mg, *V* = 100 mL, pH = 8, *τ* = 5 min. Each point is an average of three parallel determinations

Taking into account all the aforementioned data, 1-naphtol determination was performed under the following conditions of azo coupling: pH = 8, time of solid–liquid phase contact 5 min, and concentration factor equal to 2000 mL g^−1^. In the studied conditions, the calibration graph (i.e., *F*(*R*) concentration dependence) appeared perfectly linear in 0–0.8 mg L^−1^ 1-naphtol concentration range (Fig. 5); the line equation is as follows:

$$ y\kern0.5em =\kern0.5em \left(2.16\kern0.5em \pm \kern0.5em 0.03\right)x\kern0.5em +\kern0.5em \left(0.011\kern0.5em \pm \kern0.5em 0.013\right);\kern0.5em {R}^2=0.999. $$

LOD and limit of quantification (LOQ) values (20 and 66 μg L^−1^, correspondingly) were calculated from the parameters of calibration graph according to [31]. The LOD value corresponds to only 20 % of MAC for 1-naphthol in drinking water, reported in [30]; therefore, the sensitivity of the proposed method is sufficient for analysis of natural waters.

To evaluate the performance characteristics of the proposed method, model aqueous solutions of 1-naphtol were analyzed. Three parallel samples were tested for 100 and 600 μg L^−1^ solutions, and six parallel samples of as-prepared 300 μg L^−1^ solutions were analyzed twice with a 2-week period. The analysis results (confidence intervals, apparent recoveries, and RSDs) calculated according to [31] are presented in Table 3. These values demonstrate sufficient trueness, repeatability, and precision of the method and also the stability of prepared SPAR (SiO_2_–APAA) for at least 2 weeks storage.

**Table 3:** Analysis of 1-naphthol in deionized and natural water; concentration factor is 2000, *t* (*p* > 0.95, *n* = 3) = 4.303

Type of the water	1-Naphthol added (μg L^−1^)	1-Naphthol found (μg L^−1^)	Mean recovery, %	RSD, %
Deionized water	100	99 ± 11	99.0	4.6
	300*	295 ± 8	98.2	2.6
	300*	304 ± 13	101.3	4.0
	600	595 ± 31	99.2	2.1
Lake water	–	2.0 ± 6.6	–	–
	100	98 ± 6	97.7	2.6
Lake water (reference method [22])	–	2.2 ± 7.3	–	–
	100	97 ± 12	97.2	5.0

*t (*p* > 0.95, *n* = 6) = 2.571

Results of 1-naphthol analysis in the water from Lake Vyrlytsa (Kyiv), presented in Table 3, demonstrate an applicability of the proposed method for environmental water samples. The concentration of 1-naphtol in the lake water appeared below the sensitivity limits for both the proposed method and reference method described in [22]. Both tested methods give true (within the confidence interval) values of 1-naphtol concentration in the lake water spiked with MAC (100 μg L^−1^) of 1-naphtol.

To check the applicability of the proposed method for the analysis of 1-naphtol in different matrixes, the selectivity was investigated for following interferents: (i) inorganic salts and (ii) azo coupling active organic compounds. No influence of 100 mg L^−1^ of calcium, magnesium, sulfate, and carbonate ions, commonly present in environmental waters, was found. The results of 1-naphtol determination in the presence of organic interferents are shown in Table 4. Even high concentrations of phenol, resorcinol, and catechol did not interfere with 1-naphthol determination, probably due to their low activity in azo coupling with SiO_2_–DS, as discussed above. 2-Naphtol had no significant influence if present at the same concentration level as 1-naphtol analyte; however, its higher concentration resulted in a rise of the band at 490 nm in the DR-UV spectra and overestimated 1-naphtol analytical value as a result. Aromatic amines (except low-active 4-nitroaniline) also have significant interference due to their high activity in azo coupling.

**Table 4:** Effect of possible interferes on the determination of 1-naphthol, *t* test (*p* = 0.95, *n* = 3) = 4.303. Added amount of 1-naphthol is 100 μg L^−1^

Possible interferents	Molar relation of interfering reagent	1-Naphthol found, μg L^−1^
Phenol	100	99 ± 9
Resorcinol	100	96 ± 10
Catechol	50	97 ± 12
2-Naphthol	1	105 ± 11
3-Aminobenzoic acid	1	154 ± 32
Diphenylamine	10	188 ± 15
4-Nitroaniline	100	141 ± 20

## Conclusions

The *m*-aminophenylarsonic acid could be efficiently immobilized on the silica gel surface via the ion exchange with covalently grafted propyl-trimethylammonium bromide groups. The diazotation of SiO_2_–APAA in slightly acidic conditions gives immobilized diazonium salt groups stable in neutral and slightly basic media. Resulted diazonium-silica can rapidly and selectively chemisorb the azo coupling active compounds, particularly phenols, forming bright-colored azo dyes. By measuring the color intensity of resulted azo dye, concentration of 1-naphtol in aqueous solutions can be detected. The sensitivity of such solid-phase spectrophotometric procedure is sufficient for the analysis of 1-naphtol pollutant in natural waters at the level above 0.2 MAC.

## Abbreviations

DR-UV: UV diffuse reflection
DS: diazonium salt
LOD: limit of detection
LOQ: limit of quantification
MAC: maximum allowed concentration
*m*-APAA: *m*-aminophenylarsonic acid
RSD: relative standard deviation
SiO_2_–DS: silica modified with *m*-diazophenylarsonic acid
SiO_2_–*m*-АPAA: silica with covalently immobilized *m*-APAA groups
SiO_2_–TMPA: silica with covalently immobilized TMPA groups
SPAR: solid-phase analytical reagent
TMPA: trimethyl(3-trimethoxysilylpropyl)ammonium iodide
